# Making the Case and Laying the Groundwork for Integrating Age-Friendly Practices Into a University Strategic Plan

**DOI:** 10.1093/geroni/igaa057.1808

**Published:** 2020-12-16

**Authors:** Melissa Cannon

**Affiliations:** Western Oregon University, Monmouth, Oregon, United States

## Abstract

A crucial first step in preparing to become an Age-Friendly University (AFU) is seeking endorsement from the campus community and leadership. This presentation describes the mapping of the AFU principles to the strategic plan and initiatives of Western Oregon University, leading to endorsement by its faculty senate, and highlights a study of the older community members’ use of the university, laying the groundwork for advancing age-friendliness on campus. Data were collected through surveys (N=46), interviews (N=9), and photovoice method (N=7) with older adults, and data were analyzed using SPSS, team coding, and intensive group discussion to develop categories and themes. Themes emerged related to how the college campus is used by older adults, the need to promote lifelong learning to the community, and the need to address accessibility issues in order to be more age-friendly, providing helpful insight to other institutions of higher education seeking to join the AFU network.

